# The Pursuit of Self-Esteem and Its Motivational Implications

**DOI:** 10.5334/pb.277

**Published:** 2016-07-13

**Authors:** Jolene van der Kaap-Deeder, Sofie Wouters, Karine Verschueren, Veerle Briers, Bram Deeren, Maarten Vansteenkiste

**Affiliations:** 1 Faculty of Psychology and Educational Sciences, Department of Developmental, Social, and Personality Psychology, Ghent University, Ghent, Belgium; 2 Faculty of Psychology and Educational Sciences, School Psychology and Child and Adolescent Development, KU Leuven, Leuven, Belgium; 3 VCLB Oostkust, Blankenberge, Belgium

**Keywords:** contingent self-esteem, motivation, engagement, introjection, Self-Determination Theory

## Abstract

Although recent studies have found contingent self-esteem (CSE) to be negatively related to individuals’ well-being, research concerning its implications for motivation and engagement is scarce. In two studies, we investigated the relation between CSE, motivation, and engagement in achievement-related situations. A first cross-sectional study among second year high school students (*N* = 641; 54.1% female) confirmed the hypothesized motivational ambiguity associated with academic CSE. Beyond the contribution of academic self-esteem, academic CSE was positively related to behavioral and emotional engagement, but also to emotional disaffection and test anxiety. These associations could partially be explained by motivational quality, as CSE was also positively related to both autonomous and controlled types of motivation. In a second experimental study among university students (*N* = 72; 70.8% female), who participated in a tangram puzzle task under varying feedback circumstances, global CSE related to more tension, while predicting less behavioral task perseverance. These effects were not moderated by the type of feedback provided (i.e., positive vs. negative). Theoretical and practical implications of these results are discussed.

Research on self-esteem has mainly focused on people’s level of self-esteem, which entails the overall positivity or negativity towards the self (Rosenberg, Schooler, Schoenbach, & Rosenberg, 1995). Increasingly, however, other aspects of individuals’ self-esteem, including its contingency, have been found to relate to adjustment (Heppner & Kernis, 2011; Zeigler-Hill, 2013). Contingent self-esteem (CSE) denotes the extent to which people base their self-worth on meeting certain internal or external standards (Deci & Ryan, 1995) and has been studied as both a global (e.g., Kernis, 2003) and domain-specific (e.g., Crocker, Luhtanen, Cooper, & Bouvrette, 2003) characteristic. Although several studies found CSE to relate positively to maladjustment (e.g., Burwell & Shirk, 2006; Lakey, Hirsch, Nelson, & Nsamenang, 2014; Lawrence & Williams, 2013; Neighbors, Larimer, Geisner, & Knee, 2004), important gaps remain. For instance, research concerning the motivational correlates of CSE is scarce. Yet, this may be particularly interesting, as students with elevated levels of CSE may be highly motivated to do well, yet at the same time their motivation may be fraught with feelings of inner tension, anxiety, and compulsion. Hence, the present contribution aims to explore in greater detail the hypothesized motivational ambiguity comprised in CSE and whether such ambiguity would also be reflected in individuals’ emotional and behavioral engagement and disaffection in achievement settings. Specifically, Study 1 focused on the motivational, engagement and disaffection correlates of academic CSE in a sample of high school students. Study 2 sought to complement Study 1 by examining the main effect of global CSE and its interaction with type of feedback in the prediction of several motivational and engagement-related outcomes during a specific experimental task in a sample of university students.

## Contingent Self-esteem

CSE refers to individuals’ global or domain-specific tendency to hinge their self-esteem upon meeting certain internal or external standards (Deci & Ryan, 1995). Global CSE is only moderately negatively correlated with global self-esteem (e.g., Wouters, Duriez, et al., 2013). This negative interrelation suggests that individuals with lower self-esteem levels are more likely to have their self-worth interwoven with the attainment of specific standards. Paralleling this negative relation, self-esteem level and self-esteem contingency were found to yield opposite associations with adjustment, including well-being (Zeigler-Hill, 2013; Zeigler-Hill, Besser, & King, 2011), anxiety and eating disorder symptoms (Bos, Huijding, Muris, Vogel, & Biesheuvel, 2010), substance abuse (Chen, Ye, & Zhou, 2013; Tomaka, Morales-Monks, & Shamaley, 2013) and suicidal behavior (e.g., Lakey et al., 2014).

Although increasing research has indicated the detrimental effects of CSE for individuals’ adjustment, far less is known about the motivational implications of CSE. To perceive themselves as good and worthy, individuals with higher levels of CSE constantly need to reach certain goals (Zeigler-Hill, Stubbs, & Madson, 2013). However, as everyone else, they will sometimes experience failure. Because failure with regard to self-related goals is closely tied to one’s worth as a person among individuals with a high level of CSE, such failure may not be easily dismissed (Crocker & Wolfe, 2001). Hence, individuals high in CSE may be highly motivated (i.e., quantity of motivation) to pursue success (and to avoid failure) in domains in which their self-esteem is invested (Lawrence & Williams, 2013). However, the type of reasons (i.e., quality of motivation) underlying their efforts may not be completely positive as individuals high in CSE may strive to perform well for pressured reasons. In the current contribution, grounded in Self-Determination Theory (SDT; Deci & Ryan, 2000), we sought to shed a nuanced light on the different types of motives underlying the motivational functioning of individuals high in CSE.

## The Motivational Ambiguity Associated with Contingent Self-esteem

SDT distinguishes between different types of motives that fall along a continuum of increasing self-endorsement or autonomy (Deci & Ryan, 2000; Vansteenkiste, Lens, & Deci, 2006). First, individuals may be motivated out of external pressures, such as meeting demanding expectations, garnering social approval and controlling rewards or avoiding criticism. As the reason for activity engagement is situated completely outside the individual, *external regulation* is characterized by a complete lack of self-endorsement. For instance, individuals might put effort in their studies merely to please their parents. Yet, the pressure may also come from internal forces, including the avoidance of feelings of shame, guilt, and anxiety, as well as the attainment of esteem. This form of motivation has been labeled *introjected regulation* and seems especially characteristic of individuals high in CSE. Individuals who strive for an A+ to feel worthy and esteemed display introjected regulation. Although the motive is now internal to the person, the activity engagement goes along with feelings of inner conflict and compulsion as the reason for performing the activity is not fully congruent with the person’s values and convictions. Although CSE and introjected regulation are conceptually related, CSE is considered to be a relatively stable personality characteristic (Deci & Ryan, 1995) and focuses more on the link between self-esteem and performance-outcomes (i.e., success versus failure). In contrast, introjected regulation is a motivational subtype that focuses on the activity itself (i.e., why does a person undertake a certain activity?) and, although introjected regulation can be concerned with self-esteem attainment, it also includes other feelings that are more loosely related to the self (e.g., the avoidance of guilt). As both external as well as introjected regulation are characterized by pressure, these are often combined under the label of controlled motivation (e.g., Vansteenkiste, Lens, Dewitte, De Witte, & Deci, 2004).

Controlled motivation is contrasted with autonomous motivation, which also consists of at least two subtypes. Identified motivation denotes the extent to which individuals identify with the self-importance of the behavior and consider it as congruent with their own inner values. Individuals who put effort in their school work as they perceive their studies to contribute to their professional and personal development exhibit *identified regulation*. While the activity is instrumental to achieve innerly held values in the case of identified regulation, the activity constitutes a source of enjoyment and interest in itself in the case of *intrinsic motivation*. When students make their homework out of pure interest and curiosity, they are said to be intrinsically motivated. Both identified regulation and intrinsic motivation represent indicators of autonomous motivation as the reasons for engaging in the activity are fully endorsed by the individual’s self.

These different types of motives can be situated and studied at three distinct levels, that is, in relation to a specific activity at a specific moment (i.e., situational level), in a more global life domain, such as school (i.e., contextual level), or towards life in general (i.e., global level) (Vallerand, 1997). In this study, we focused on motivation at the situational and contextual level. Previous research has provided evidence for the beneficial effects of autonomous study motivation and the fairly detrimental effects of controlled study motivation among diverse populations, differing in age, gender, and cultural background (Deci & Ryan, 2000; Lens & Vansteenkiste, 2006; Vansteenkiste & Ryan, 2013). Specifically, both intrinsic motivation (Taylor et al., 2014) and identified motivation (Burton, Lydon, D’Alessandro, & Koestner, 2006) have been found to relate positively to school performance and persistence, while relating to less procrastination among (pre-)college students (Vansteenkiste, Zhou, Lens, & Soenens, 2005). In contrast, controlled motivation has been found to relate negatively to persistence and efficacious time planning among high school and college students (e.g., Michou, Vansteenkiste, Mouratidis, & Lens, 2014; Ratelle, Guay, Vallerand, Larose, & Senecal, 2007), while being positively related to test anxiety among high school students (Vansteenkiste, Sierens, Soenens, Luyckx, & Lens, 2009).

Theoretically, CSE is assumed to relate primarily to introjected regulation (Crocker & Wolfe, 2001; Deci & Ryan, 1995; Kernis, 2003), although evidence for this claim is scarce and rather indirect. Specifically, previous experimental work on the effect of task- versus ego-involvement on autonomy and intrinsic motivation (e.g., Ryan, 1982) is relevant. In these studies, participants’ ego or self-esteem was primed prior to engaging in a specific task by suggesting the task is indicative of their creative or social intelligence. In doing so, participants’ task-specific CSE was temporarily activated. Ryan, Koestner, and Deci (1991) found that the induction of ego-involvement relative to task-involvement undermined college students’ sense of choice and autonomy. Moving beyond past work, the present study investigated the relation between CSE and the various types of motives as discerned within SDT by directly assessing these constructs. Although individuals high in CSE would regulate their learning behavior primarily on the basis of introjection, the attachment of their self-worth to the outcome of their functioning may also lead them to value the learning more (i.e., identified regulation). At the same time, the tension underlying their function may shift away their focus from the learning itself and preclude them to fully enjoy and get interested in the learning, thus potentially undermining their intrinsic motivation. Finally, Wouters, Doumen, Germeijs, Colpin, and Verschueren (2013) found that psychologically controlling parenting (i.e., characterized by pressure from parents on children to think, feel, or act in certain ways) related to higher levels of CSE among early adolescents. Therefore, individuals high in CSE may be motivated more out of perceived external pressure.

## The Engagement Ambiguity Associated with Contingent Self-esteem

In analogy to the presumed motivational ambiguity characteristic of CSE, we expected a similar mixed pattern for engagement. In line with the multidimensional nature of engagement (Fredericks, Blumenfeld, & Paris, 2004; Skinner, Furrer, Marchand, & Kindermann, 2008), this study focused on four important dimensions of classroom engagement: (1) behavioral engagement, (2) emotional engagement, (3) behavioral disaffection, and (4) emotional disaffection. Engagement refers to adaptive motivational states with behavioral engagement referring to effort exertion and persistence during learning, on-task behavior and participation in learning activities and emotional engagement referring to positive and energized emotions during learning (e.g., enthusiasm, interest and enjoyment; Skinner, Kindermann, & Furrer, 2009). Disaffection, on the contrary, reflects the presence of maladaptive motivational states and, as such, this concept is broader than the sheer absence of engagement. Analogous with engagement, disaffection also consists of two components: behavioral disaffection (e.g., passive behavior during learning activities) and emotional disaffection (e.g., negative emotions such as anxiety; Skinner et al., 2008). Previous research has found engagement and disaffection to yield, respectively, a positive and negative relation to school success (Skinner et al., 2008).

To the best of our knowledge, there are currently no studies directly linking CSE to behavioral or emotional engagement and disaffection. However, there is some research pointing to links between CSE and variables related to engagement. Concerning behavioral engagement, the higher students’ levels of academic CSE the more hours they spent studying and looking at solutions to analytical problems they had to solve (Crocker, Brook, Niiya, and Villacorta, 2006; Crocker, Luhtanen, et al., 2003). Yet, the perseverance of individuals high in CSE may be shaky and conditional. Indeed, the experimental induction of ego-involvement (i.e., a momentary state of CSE), only resulted in continued behavioral persistence during a free-choice period if participants received no or non-confirmative feedback, while the persistence faded if they had received positive feedback (Ryan et al., 1991). Presumably, prompting participants’ ego only fosters persistence when participants’ desired outcome (i.e., performing successfully as indicated by positive feedback) is not yet achieved, while their persistence wanes quickly if they do achieve their desired outcome, underscoring its conditional character. Consistent with this interpretation, Van Wijhe, Peeters, and Schaufeli (2014) showed that individuals whose self-esteem highly depends on work achievements, felt compelled by themselves to work hard, being indicative of persistence, yet of the internally pressuring sort (see also Van den Broeck, Schreurs, De Witte, Vansteenkiste, Germeys, & Schaufeli, 2011). Overall then, individuals high in CSE would display a mix of both behavioral engagement and disaffection.

Furthermore, with regard to emotional disaffection, Lawrence and Williams (2013) demonstrated that, in an evaluative setting, undergraduate students with higher levels of academic CSE reported higher levels of test anxiety. Further, students high on academic CSE were found to suffer more from bad grades in terms of self-esteem and positive affect than they benefitted from good grades, indicating that these students are more sensitive to failure in the academic domain and thus may be more anxious in an achievement setting (Crocker, Karpinski, Quinn, & Chase, 2003).

## The Present Research

The primary aim of the present contribution was to examine the motivational and engagement correlates of CSE. Overall, because individuals high in CSE would be highly committed to the activity yet also emotionally more tense and conflicted about their activity engagement, we expected CSE to relate to a mixed pattern of motivational and engagement outcomes. An additional aim was to explore the conditions under which CSE would especially yield a harmful or rather benign effect by examining whether (a) there is an optimal point in CSE (i.e., curvilinear relation), (b) effects of CSE would depend on level of self-esteem (i.e., an interaction effect), and (c) the harmful correlates of CSE would especially become salient under negative feedback circumstances.

To this end, a cross-sectional and an experimental study were conducted among, respectively, high school and university students. Whereas Study 1 focused on the contextual level of CSE, motivation, and engagement (i.e., school) and focused on a variety of engagement and disaffection indicators, Study 2 focused on the situational level (i.e., task-specific) with regard to motivation and engagement, involved an experimental manipulation of feedback type (i.e., positive or negative), and included an objectively recorded indicator of behavioral engagement. Further, while Study 1 focused on academic CSE, Study 2 included a measure of global CSE. We chose to assess global CSE in Study 2, as the task (i.e., puzzle task) did not directly relate to a specific domain (e.g., academic, social).

## Study 1

Study 1 was conducted among a large sample of high school students in their second year. We chose to focus on these students as they need to make important track and subject choices when transitioning from Grade 8 to Grade 9. Apart from including diverse motivational and engagement subtypes, we also included a separate measure for test anxiety, which has been found to yield various negative consequences for students’ learning and performance (Zeidner & Matthews, 2005). The following set of three hypotheses and two research questions was formulated. First, the hypothesized motivational ambiguity of CSE would manifest through its positive relation with introjected, identified regulation and external regulation, and a negative relation with intrinsic motivation (Hypothesis 1). That is, while CSE would relate primarily to an internally pressuring form of regulation (i.e., introjected), it would also relate to identified regulation, as CSE goes together with a high commitment to the activity (Crocker, Luhtanen, et al., 2003; Lawrence & Williams, 2013). Further, as CSE is characterized by the feeling that one’s worth is at stake in achievement-settings, we expected that this tension may preclude individuals from fully enjoying learning (i.e., a negative relation between CSE and intrinsic motivation). Finally, as CSE has been found to relate to controlling parenting, individuals high in CSE may pursue academic goals more out of perceived external pressures (i.e., external regulation).

Second, the hypothesized engagement ambiguity of CSE would manifest itself through its positive relation with behavioral engagement and emotional disaffection, and a negative relation with emotional engagement (Hypothesis 2). That is, although individuals high in CSE would be inclined to display a high level of behavioral engagement to preserve or increase their self-worth, their activity engagement would come with feelings of pressure, (test) anxiety, and frustration, thus coming along with emotional disaffection. We further expected that these negative feelings would be so heavily present that individuals high in CSE would fail to display any emotional engagement. It is unclear whether these dynamics would also apply to individuals’ behavioral disaffection. Hence, the relation with behavioral disaffection was examined exploratively.

Third, the simultaneous inclusion of both motivational and engagement variables allowed us to examine whether motivation would account for (i.e., mediate) the relation between CSE and engagement (Hypothesis 3). For instance, the hypothesized positive relation between CSE and emotional disaffection may be carried by introjected regulation, while the hypothesized positive relation with behavioral engagement may be carried by identified regulation. Finally, we explored whether CSE would be less harmful when individuals experienced a moderate level of CSE (as opposed to a low or high level) (Research Question 1). More specifically, a low level of CSE could be regarded as an indicator of a careless attitude, whereas a high level of CSE might cause an overdose of internal pressure. In other words, a moderate level of CSE would represent the ideal point. Further, we explored whether the link between CSE and motivation/engagement was moderated by the level of self-esteem (Research Question 2). Some previous studies found evidence for this notion with the detrimental effects of CSE being especially pronounced when combined with low levels of self-esteem (e.g., Bos et al., 2010), whereas others did not (e.g., Wouters, Duriez, et al., 2013). To investigate the unique effects of CSE above and beyond the level of self-esteem, we always controlled for level of self-esteem in both studies.

## Method

### Participants and Procedure

Several high schools in the Dutch-speaking part of Belgium were invited to participate in a study examining students’ transition from Grade 8 to Grade 9. Seventy schools were chosen to ensure representativeness with regard to educational network (private (mainly Catholic) versus public education), geographical location and educational level; 35 schools eventually agreed to participate. From each school, one class was randomly selected to participate. Before students filled out the questionnaire, they signed a standard consent form which informed them that they could refuse or discontinue participation at any time. A total of 641 students agreed to participate; passive parental consent for all these students was obtained a few weeks prior to the study. Students completed online questionnaires in the computer room of their school during a collective session supervised by Psychology bachelor students. The survey was divided into two parts, which were presented in random order to students belonging to the same class as to avoid order effects. The mean age in this sample was 14.06 years (*SD* = 0.64; range 10 to 17 years; 11.1% missing values), with most participants being female (54.1% female and 2.3% missing values).

### Measures

All items were answered in Dutch on a Likert-type scale ranging from 1 (*Does not apply to me at all*) to 5 (*Completely applies to me*), unless indicated otherwise.

**Academic Contingent Self-Esteem.** Academic CSE was measured with four items from the academic subscale of the Self-Worth Contingency Questionnaire (Burwell & Shirk, 2003; Wouters, Doumen, et al., 2013). We selected these four items from the total 8-item subscale because they had the highest loadings on the factor academic CSE in another dataset consisting of adolescents (Wouters, Doumen, et al., 2013). A sample item is: “Whether or not I reach my goals in school strongly affects my feelings of worth”. Internal consistency of the scores was good (α = .87).

**Academic Self-Esteem.** To measure academic self-concept, students filled out three items from an adapted subscale of the Self-Description Questionnaire (SDQ) II (Marsh, 1992). A sample item is: “Most school subjects are just too hard for me”. Internal consistency was sufficient (α = .66).

**Motivation.** The quality of students’ motivation in the academic domain (i.e., their motives for studying) was assessed with the Dutch adapted version (Vansteenkiste et al., 2009) of the Academic Self-Regulation Questionnaire (SRQ-A) developed by Ryan and Connell (1989). This questionnaire measures four motivational types with each four items: intrinsic motivation (“I’m studying because I enjoy doing it”; α = .86), identified regulation (“I’m studying because this is an important life goal to me”; α = .79), introjected regulation (“I’m studying because I would feel guilty if I wouldn’t do so”; α = .70), and external regulation (“I’m studying because others (parents, friends, etc.) force me to do this”; α = .68).

**Engagement.** Students’ levels of behavioral and emotional engagement and disaffection were measured with the Dutch translated version (Verschueren & Wouters, 2012) of the engagement scales developed by Skinner et al. (2008). Sample items are “I try hard to do well in school” (behavioral engagement; 5 items; α = .83), “I enjoy learning new things in class” (emotional engagement; 5 items; α = .83), “In class, I do just enough to get by” (behavioral disaffection; 5 items; α = .80), and “When I’m doing work in class, I feel bored” (emotional disaffection; 12 items; α = .82). Items were rated on a scale ranging from 1 (*Not true at all*) to 4 (*Completely true*). All subscales were internally consistent.

**Test anxiety.** Test anxiety was assessed with a subscale consisting of eight items from a Dutch questionnaire concerning study management abilities (Depreeuw & Lens, 1998). A sample item is “During the school year, I feel very tense when I study”. This scale was internally consistent (α = .81).

### Plan of Analyses

For the present set of main variables, only 2.79% of the data at the scale level were missing. Participants with and without complete data were compared using Little’s (1988) Missing Completely At Random (MCAR) test. This resulted in a normed chi square (i.e., χ²/*df*) of 1.69, which suggests that values were missing at random. Hence, we used the Full Information Maximum Likelihood (FIML) procedure (Schafer & Graham, 2002) in Mplus 6.1 (Muthén & Muthén, 1998–2010). Standard fit indices were used to evaluate all models. For adequate fit, the Root Mean Square Error of Approximation (RMSEA) and the Standardized Root Mean square Residual (SRMR) less than or equal to .08, the Comparative Fit Index (CFI) equal to or larger than .90; acceptable fit was also indicated by a χ²/*df* ratio of 2 or below (Hu & Bentler, 1999; Kline, 2005). To test the significance of indirect effects, we used bootstrapping (using 1,000 draws), a nonparametric resampling procedure that is highly recommended (Preacher & Hayes, 2008). We did not consider multilevel modeling or controlling for clustering in schools for several reasons: (1) the average ICC was very small .03 (ICC’s ranged from .00 to .06)^1^, (2) all design effects were below 2, and (3) we had no variables available at the school level (Peugh, 2010).

## Results

### Descriptive Statistics and Preliminary Analyses

Table 1 presents all means, standard deviations, and intercorrelations. The level and the contingency of self-esteem were slightly positively correlated and they were positively related to all types of motivation, except for the negative correlation between academic self-esteem and external regulation. Further, contingency and level of SE were both positively related to behavioral and emotional engagement, while being negatively related to behavioral disaffection. Additionally, academic CSE was positively related to emotional disaffection and test anxiety, whereas academic self-esteem was negatively related to these outcomes. As for the motivational variables, their intercorrelations followed a simplex pattern, with motivational subtypes being closer to each other on the continuum of increasing autonomy (e.g., intrinsic and identified) being more strongly correlated than subtypes being positioned further away (e.g., intrinsic and external). This simplex pattern was also evident in the pattern of correlations between the motivational subtypes and the various engagement indicators and text anxiety: The two autonomous forms of regulation were similarly related to engagement (positively) and disaffection (negatively), whereas external regulation was only positively related to disaffection. The correlates of introjection, the subtype situated between identified and external regulation, sometimes mirrored those of autonomous regulations and sometimes those of external regulation. Although all motivational subtypes related positively to test anxiety, this relation was stronger for the two controlled forms of regulation.

**Table 1:** Descriptives of and Correlations between the Variables (Study 1).

	1	2	3	4	5	6	7	8	9	10	11

1. Academic CSE	–										
2. Academic SE	.09*	–									
3. Intrinsic motivation	.35***	.26***	–								
4. Identified regulation	.39***	.33***	.67***	–							
5. Introjected regulation	.41***	.14***	.41***	.47***	–						
6. External regulation	.19***	–.11**	–.06	.02	.31***	–					
7. Behavioral engagement	.31***	.31***	.33***	.44***	.29***	.03	–				
8. Emotional engagement	.22***	.30***	.42***	.43***	.20***	–.05	.53***	–			
9. Behavioral disaffection	–.11**	–.33***	–.32***	–.37***	–.20***	.15***	–.40***	–.28***	–		
10. Emotional disaffection	.18***	–.29***	–.10*	–.14***	.08*	.20***	–.12**	–.22***	.52***	–	
11. Test anxiety	.41***	–.31***	.13**	.13**	.25***	.20***	.05	–.03	.15***	.47***	–

*M*	2.97	3.63	2.17	3.40	2.77	2.96	3.12	2.75	2.17	2.13	2.76
*SD*	1.04	0.76	0.90	0.94	0.92	0.86	0.52	0.64	0.63	0.50	0.82

*Note.* CSE = Contingent self-esteem, SE = Self-esteem. *p < .05. **p < .01. ***p < .001.

Finally, significant sex differences were found with an independent samples *t*-test (with Levene’s test for testing the equality of the variances) for academic CSE, *t* (616.72) = –2.47, *p* < .05, identified regulation, *t* (624) = –2.47, *p* < .05, behavioral disaffection, *t* (602) = 2.91, *p* < .01, and test anxiety, *t* (620) = –2.67, *p* < .01. More specifically, girls reported more academic CSE (*M* = 3.06, *SD* = 1.09), more identified regulation (*M* = 3.48, *SD* = 0.94), more test anxiety (*M* = 2.84, *SD* = 0.81), and less behavioral disaffection (*M* = 2.10, *SD* = 0.60) than boys (*M* = 2.86, *SD* = 0.98, *M* = 3.30, *SD* = 0.93, *M* = 2.67, *SD* = 0.83, and *M* = 2.25, *SD* = 0.67 respectively). In light of these gender effects, gender was controlled for in all models (only significant gender effects were retained).

### Primary Analyses

In a first model^2^, we looked at the unique contribution of academic CSE and academic self-esteem in the prediction of all four motivation types (all motivation types were allowed to correlate). The fit of this model was excellent (χ²/*df* = 6.23/5 = 1.25, *p* = .28, RMSEA = .02, 90% CI RMSEA [.00–.06], CFI = 1.00, SRMR = .01, *N* = 626). Controlling for academic self-esteem level, results showed that academic CSE was positively related to all types of motivation (β*_intrinsic_* = .33, *p* < .001; β*_identified_* = .37, *p* < .001; β*_introjected_* = .40, *p* < .001; β*_external_* = .20, *p* < .001).

In two following models, we investigated the unique contribution of academic CSE and academic self-esteem to indicators of, respectively, engagement and disaffection (all outcomes were allowed to correlate in each model). Model fit was good in both models (engagement model: χ²/*df* = 2.49/3 = 0.83, *p* = .48, RMSEA = .00, 90% CI RMSEA [.00–.06], CFI = 1.00, SRMR = .01, *N* = 626; disaffection model: χ²/*df* = 3.72/2 = 1.86, *p* = .16, RMSEA = .04, 90% CI RMSEA [.00–.10], CFI = 1.00, SRMR = .01, *N* = 626). Controlling for self-esteem level, CSE was positively related to behavioral (β = .28, *p* < .001) and emotional engagement (β = .19, *p* < .001). Further, CSE was unrelated to behavioral disaffection (β = –.07, *p* = .06), but positively related to emotional disaffection (β = .22, *p* < .001) and test anxiety (β = .44, *p* < .001).

**Figure 1:**
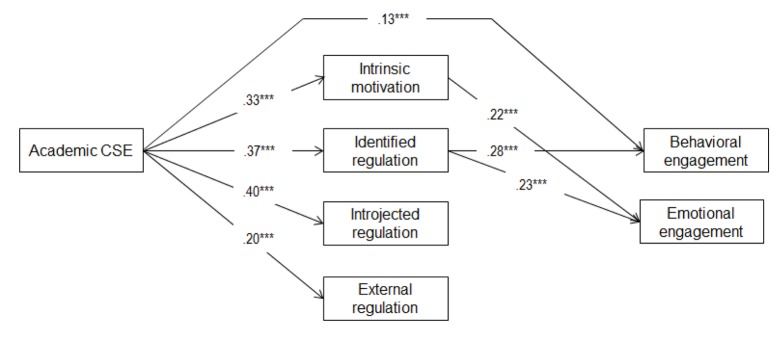
Structural Model Depicting the Relation between Contingent Self-esteem, Motivation, and Engagement (Study 1). *Note.* Standardized path coefficients are shown. Only significant paths are shown. Direct relations of self-esteem level with mediators and outcomes, covariances between self-esteem level and contingency, covariances between our 4 mediators, covariances between our outcomes and significant gender effects were estimated, but not shown for clarity purposes. *p < .05.**p < .01.***p < .001.

**Figure 2:**
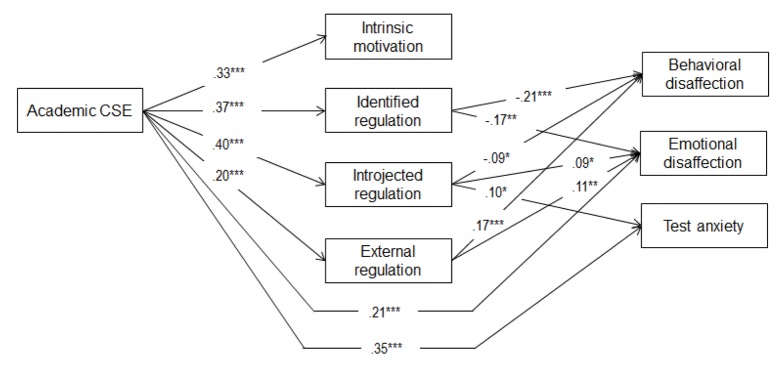
Structural Model Depicting the Relation between Contingent Self-esteem, Motivation, and Disaffection (Study 1). *Note.* Standardized path coefficients are shown. Only significant paths are shown. Direct relations of self-esteem level with mediators and outcomes, covariances between self-esteem level and contingency, covariances between our 4 mediators, covariances between our outcomes and significant gender effects were estimated, but not shown for clarity purposes. *p < .05.**p < .01.***p < .001.

In a third step, we estimated two mediational models with the four types of motivation mediating the effect of both self-esteem aspects on engagement and disaffection respectively; direct paths from the self-esteem aspects on engagement and disaffection were added in block and only retained if they were significant. This resulted in the two final models as shown in Figures 1 and 2. The fit of these models was excellent (engagement model: χ²/*df* = 9.95/8 = 1.24, *p* = .27, RMSEA = .02, 90% CI RMSEA [.00–.05], CFI = 1.00, SRMR = .02, *N* = 626; disaffection model: χ²/*df* = 12.95/8 = 1.62, *p* = .11, RMSEA = .03, 90% CI RMSEA [.00–.06], CFI = 1.00, SRMR = .02, *N* = 626). The associations between CSE and motivation were similar to those in Model 1: All relations were significantly positive. The direct relations between CSE and engagement and disaffection were also similar to those in Models 2 and 3, except for the direct association between academic CSE and emotional engagement which was no longer significant when the mediators were added.^3^

Next, we performed bias corrected bootstrapping in Mplus with 1,000 draws and controlling for self-esteem level, we found several significant indirect relations between CSE and engagement and disaffection in the final models. These indirect relations are summarized in Table 2. Results yielded a positive indirect relation between academic CSE and engagement through its positive association with identified regulation and/or intrinsic motivation. Indirect relations with disaffection were more inconsistent in that negative relations were found through identified regulation, positive relations via external regulation, and both positive and negative relations through introjected regulation.

**Table 2:** Overview of all Significant Indirect Effects from Academic Contingent Self-Esteem to all Outcomes in Models 4 and 5 (Study 1).

Indirect paths					95% CIs

ACSE	→	Identified regulation	→	Behavioral engagement	[0.058, 0.149]
ACSE	→	Intrinsic motivation	→	Emotional engagement	[0.038, 0.109]
ACSE	→	Identified regulation	→	Emotional engagement	[0.039, 0.133]
ACSE	→	Identified regulation	→	Behavioral disaffection	[–0.124, –0.033]
ACSE	→	Introjected regulation	→	Behavioral disaffection	[–0.068, –0.002]
ACSE	→	External regulation	→	Behavioral disaffection	[0.011, 0.056]
ACSE	→	Identified regulation	→	Emotional disaffection	[–0.111, –0.012]
ACSE	→	Introjected regulation	→	Emotional disaffection	[0.002, 0.072]
ACSE	→	External regulation	→	Emotional disaffection	[0.004, 0.041]
ACSE	→	Introjected regulation	→	Test anxiety	[0.007, 0.077]

*Note.* ACSE = Academic contingent self-esteem.

### Supplementary Analyses

Finally, in a set of supplementary analyses (in which both self-esteem aspects were standardized), we tested for quadratic effects of academic CSE in Models 1 to 3. Results showed that only 2 out of 9 quadratic terms related significantly to the outcomes (βs ranged from –.16 to .00). Additionally, we tested for significant interaction effects between academic CSE and academic self-esteem in Models 1 to 3. Results showed that only 1 out of 9 interactions was significant (βs ranged from –.10 to .07)^4^. Hence, we did not include any of these effects in our final models.

### Brief Discussion

In Study 1, we found that CSE related to all types of motivation, indicating that individuals with CSE are strongly motivated to put effort in their studies. Yet, this undifferentiated pattern of correlates equally points to the ambiguous character of CSE. Although academic CSE was positively related to both intrinsic motivation and identified regulation, it was also positively related to introjected and external regulation, beyond any effects of academic self-esteem. Similarly, results showed that academic CSE related to more behavioral and emotional engagement, but also to more emotional disaffection and test anxiety. Furthermore, the significant direct contribution of CSE to emotional engagement was fully explained by the quality of students’ motivation, whereas motivational processes could only partially explain the contribution of CSE to the other engagement indicators. Specifically, academic CSE yielded an indirect positive contribution to engagement via identified regulation and/or intrinsic motivation, but was also characterized by a mixed set of positive and negative indirect effects on disaffection via a combination of different motivational types. Finally, no systematic evidence was obtained for quadratic effects or moderation by level of self-esteem.

## Study 2

Study 2 aimed to extend Study 1 in three significant ways. First, instead of investigating the role of CSE at the contextual level, we examined its role in a specific achievement-related situation, that is, when university students were working on a puzzle task during which their ego-involvement was primed. We aimed to explore whether the hypothesized motivational and engagement ambiguity, for which we found evidence in Study 1, would also manifest itself when participants completed a specific activity. Second, while Study 1 was limited to self-reports, Study 2 included an unobtrusive measure of behavioral engagement. That is, participants were given the choice to (dis)continue their participation during a free-choice period (Deci, 1971). In addition, we tapped their reasons for continuing to spent time on the target activity during this period. Third, Study 2 was experimental in nature as type of feedback (i.e., positive or negative) was varied. This allowed us to explore whether the hypothesized ambiguity of CSE would be limited to a circumstance of negative feedback or would appear across types of feedback. Apart from behavioral engagement, Study 2 also included an assessment of tension, reflecting emotional disaffection, and enjoyment, constituting emotional engagement.

We formulated the same set of hypotheses and research questions as in Study 1. During the experimental phase, we expected that higher levels of CSE would relate to more felt tension (as an indicator of emotional disaffection) as experienced during a puzzle task. Although we found a positive relation between CSE and emotional engagement in Study 1, we anticipated that CSE would relate negatively to enjoyment when individuals’ ego would be prompted (which was the case in this study) as this would foster a focus on gaining self-approval instead of enjoying the task. Further, although CSE was found to relate to greater behavioral engagement in Study 1, it remains to be seen whether this effect would get replicated when using an objective parameter, which was gathered when the experimental task was completed. That is, there was no obligation to continue with the target activity. To the extent that CSE would relate to more time spent on puzzling during a free-choice period, we expected that their persistence would be driven mainly by introjection and, in line with the results of Study 1, identification. Finally, in analogy with Study 1, in an explorative way we examined whether the proposed relations between CSE and the outcomes was moderated by the level of self-esteem and whether these relations were purely linear or rather quadratic in nature. Moreover, the experimental variation of type of feedback allowed us to examine whether the effects of CSE would be feedback-dependent or not.

## Method

### Participants and Procedure

Individuals were invited via an online participant panel system to participate in this study in return for course credits. In total, 72 individuals (of which 51 were female) participated. Participants were aged between 17 and 50 years (*M*age = 19.54; *SD* = 3.92) and were mostly first year bachelor students in psychology (*N* = 59). Furthermore, participation was voluntary and all data were processed confidentially. At the start of the study, participants gave their written consent. The study consisted of five consecutive parts: (1) filling out questionnaires concerning demographics, global CSE, and global self-esteem; (2) performing a puzzle task in either a success or failure condition; (3) filling out puzzle task-related questionnaires (i.e., felt tension, competence, and enjoyment during the puzzle task); (4) a free-choice period; and (5) filling out questionnaires concerning the motives underlying their persistence in the free-choice period. With respect to the puzzle task, participants were randomly assigned to either the success condition or the failure condition. In both conditions, ego-involvement was induced at the beginning of the activity by describing the puzzle task as a test of competence with regard to visual information processing (which resembles the ego-involvement induction as used by Ryan et al., 1991). In doing so, participants’ task-specific CSE was temporarily activated. Next, the Tangram Puzzle Task (TPT) was introduced.

**The Tangram Puzzle Task.** The TPT consists of seven geometrically different pieces that need to be correctly assembled to form specific homogeneous black figures. First, the experimenter informed participants about the puzzle task and demonstrated how to assemble the pieces to form a specific figure. Then, all participants started with the practice phase in which they were given four minutes to assemble two figures (one easy and one fairly difficult figure). This was followed by the test phase, during which participants needed to solve five puzzles. A success and failure condition were created by varying the standard of success and the level of difficulty of the figures. Specifically, in the success and failure condition, individuals were informed that 50% of their peers could, respectively, correctly assemble *two* and *four* figures. Additionally, the figures of individuals in the failure condition were more difficult, further increasing the likelihood of failing to attain the provided standard. During both the practice and test phase, participants were instructed to write down whether they had successfully assembled the puzzle before continuing with the next puzzle. After the practice phase, but before the test phase, the experimenter left the room to go to the adjacent room to observe participants through a one-way mirror. After the test phase, the experimenter reentered the room and provided the participants with the condition-specific feedback. After completing a set of questionnaires, the experimenter informed the participants that the next participant was waiting for her in the adjacent room. She asked the participants to wait a few minutes till she got back. The experimenter also explained that during this free-choice period the participants were free to do whatever they wanted, including solving some of the unresolved puzzle tasks of the experimental phase (to which we will refer as ‘old puzzles’), reading a magazine (which the experimenter was previously reading while the participant filled out questionnaires), or doing some new puzzle tasks (to which we will refer as ‘new puzzles’), which were already on the table and were said to be from another study. Subsequently, the experimenter went to the adjacent room to observe (through the one-way mirror) whether the participants continued to spent time on the puzzles or on another activity. After seven minutes, the experimenter returned to the room. If participants worked on puzzles during the free-choice period, they were provided with a questionnaire assessing the motivation for doing so. Finally, participants were debriefed and asked not to discuss the content of the study with fellow students.

### Measures

**Global Contingent Self-Esteem.** Global CSE was assessed with the Dutch version (Soenens & Duriez, 2012) of the Contingent Self-esteem Scale (CSS; Paradise & Kernis, 1999). We chose to assess global CSE (as opposed to academic CSE) as the experimental task related to achievement in a broad sense (broader than the academic domain). The CSS consists of 15 items (e.g., “I consider performing well as important for my self-esteem”) which were rated on a scale from 1 (*Not at all true*) to 5 (*Completely true*). The scores for this scale were internally consistent (α = .87).

**Global Self-Esteem.** The Dutch version (Franck, De Raedt, Barbez, & Rosseel, 2008) of the Rosenberg Self-Esteem Scale (RSES; Rosenberg, 1979) was used to assess global self-esteem. This scale consists of 10 items (e.g., “On the whole, I am satisfied with myself”) that were rated on a scale ranging from 0 (*strongly disagree*) to 3 (*strongly agree*). The internal consistency of the scores was good (α = .83).

**Puzzle Task-related Outcomes.** Three subscales of the Intrinsic Motivation Inventory (Ryan, 1982; Ryan et al., 1991) were employed, that is, (a) felt tension (2 items; e.g., “I felt very tense when making the puzzles”; α = .79), which served as an indicator of emotional disaffection, (b) perceived competence (2 items; e.g., “I think I knew well how I could make the puzzles” α = .84), which was used as a manipulation check, and (c) experienced enjoyment (5 items; e.g., “Making the puzzles was fun” α = .83) while performing the puzzle task, which served as an indicator of emotional engagement. All items were rated on a scale from 1 (*Not at all true*) to 7 (*Completely true*).

**Persistence during the Free-choice Period.** Persistence was conceptualized as the time participants spent on puzzling during the free-choice period which was recorded and expressed in seconds.

**Motivation during the Free-choice Period.** Because participants could have various motives to continue working on the puzzles during the free-choice period, their reasons for continued perseverance at both the old puzzles (i.e., those being provided during the experimental phase) and new (i.e., newly offered at the beginning of the free-choice period) were assessed. Specifically, adapting a previously used questionnaire (Vansteenkiste, Simons, Lens, Sheldon, & Deci, 2004) to assess motivation for reading a text, participants rated reasons for puzzling with 4 items assessing each type of motivation: intrinsic motivation (e.g., “Because I found this activity enjoyable”), identified regulation (e.g., “Because I found this useful”), introjected regulation (e.g., “Because I had to prove to myself that I am a good puzzler”) and external regulation (e.g., “Because I felt that others obliged me to do so”). All items were rated on a scale from 1 (*strongly disagree*) to 5 (*strongly agree*). As the subscales of the questionnaire referring to the old puzzles were highly correlated with the corresponding subscales of the questionnaire referring to the new puzzles (*r* ranging between .85 and 1.00), responses across the two questionnaires were averaged. All subscales were found to be internally consistent (intrinsic motivation: α = .86; identified regulation: α = .79; introjected regulation: α = .78; external regulation: α = .86).

## Results

### Descriptive Statistics and Preliminary Analyses

Descriptives of and bivariate correlations among the study variables are displayed in Table 3. CSE was negatively related to self-esteem. Whereas CSE related positively to tension, self-esteem was negatively related to this construct. Both were unrelated to felt competence and enjoyment during task execution and did not correlate with the time spent puzzling during the free-choice period. To the extent individuals high in CSE were persistent, they did so for a combination of introjected and identified reasons. In contrast, self-esteem related negatively to both introjected and identified regulation. As for the background variables, age was not related to any of the study variables, whereas gender was significantly related to CSE and time spent on puzzling during the free-choice period: men (*M* = 3.40; *SD* = .45) reported less CSE than women (*M* = 3.68; *SD* = .52); *t*(70) = 2.29; *p* < .05, while they (*M* = 301.86; *SD* = 154.98) persisted longer than women (*M* = 166.82; *SD* = 187.90); *t*(44.95) = –3.15; *p* < .01 (the *df* were adapted as Levene’s test indicated unequal variances between the conditions). Therefore, when predicting persistence we controlled for gender in all subsequent analyses.

**Table 3:** Descriptives of and Correlations between the Variables (Study 2).

	1	2	3	4	5	6	7	8	9	10

1. Global CSE	–									
2. Global SE	–.48***	–								
Puzzle task										
3. Competence	–.13	.10	–							
4. Tension	.36**	–.27*	–.42***	–						
5. Enjoyment	–.09	–.02	.53***	–.19	–					
Free-choice period										
6. Persistence	–.21	.01	.16	–.11	.41***	–				
7. Intrinsic motivation	.09	.02	–.00	–.05	.62***	.22	–			
8. Identified regulation	.47**	–.52***	.01	.30*	.23	.17	.17	–		
9. Introjected regulation	.48**	–.49**	–.03	.31*	–.18	.28	–.11	.51***	–	
10. External regulation	–.05	.01	.21	–.06	–.09	.17	–.20	–.23	.34*	–

*M*	3.59	1.96	2.78	4.28	4.44	206.21	3.88	3.01	2.66	1.63
*SD*	0.48	0.37	1.57	1.40	1.13	188.28	0.69	0.89	0.96	0.69

*Note.* CSE = Contingent self-esteem, SE = Self-esteem. Correlational analyses pertaining to the free-choice period variables are based on a subset of the data, as only a limited number of participants persevered (i.e., 45/72). *p < .05. **p < .01. ***p < .001.

Subsequently, we employed the Kolmogorov-Smirnov test, which is mainly suitable for small samples, to investigate whether the scores on the study variables were not significantly different from a normal distribution (i.e., the null hypothesis). The results indicated that the distribution of the scores on self-esteem (*D* (72) = .13), competence (success condition: *D* (36) = .16; failure condition: *D* (36) = .25), persistence (success condition: *D* (36) = .19; failure condition: *D* (36) = .32), and external regulation (success condition: *D* (27) = .22; failure condition: *D* (18) = .25) were significantly non-normal (all *p*s < .05). Scores on other variables were normally distributed.

To examine the condition-effects we performed two sets of MANOVAs (see Table 4), one involving the task-related outcomes and another involving participants’ reasons for persisting during the free-choice period as only a limited number persevered (i.e., 45/72). As MANOVAs are rather robust with respect to non-normality, we employed parametric MANOVAs. Individuals in the failure condition solved fewer test puzzles, reported less competence (which indicates that the manipulation was effective) as well as less enjoyment, and felt more tense than individuals in the success condition. Although condition did not affect participants’ degree of persistence, it did impact the number of individuals engaging in the puzzle activity, with fewer participants in the failure condition (50% vs. 75% in the success condition) getting engaged in the puzzling at all; c^2^ (1, *N* = 72) = 4.80, *p* = .05. The second MANOVA indicated that the success-failure manipulation did not relate to participants’ motives for persisting during the free-choice period.

**Table 4:** Comparison of the Means between the Success Condition and the Failure Condition (Study 2).

	Success condition (*N* = 36)	Failure condition (*N* = 36)	Comparison conditions	

	*M* (*SD*)	*M* (*SD*)	*F*-value	η^2^

Puzzle task				
Nr. solved practice puzzles	.92 (.73)	1.00 (.76)	*F*(1, 70)= .23	.00
Nr. solved test puzzles	3.06 (.75)	.86 (.96)	*F*(1, 70) = 116.25***	.62
Competence	3.85 (1.33)	1.72 (.94)	*F*(1, 70) = 61.09***	.47
Tension	3.93 (1.25)	4.63 (1.47)	*F*(1, 70) = 4.65*	.06
Enjoyment	4.86 (.85)	4.03 (1.22)	*F*(1, 70) = 10.91**	.14

Free-choice period				
Persistence	237.33 (179.75)	175.08 (193.93)	*F*(1, 70) = 2.00	.03
Intrinsic motivation	3.83 (.74)	3.96 (.63)	*F*(1, 43) = .37	.01
Identified regulation	3.05 (.86)	2.94 (.95)	*F*(1, 43) = .15	.00
Introjected regulation	2.75 (.99)	2.54 (.91)	*F*(1, 43) = .53	.01
External regulation	1.74 (.78)	1.47 (.48)	*F*(1, 43) = 1.68	.04

*Note.* *p < .05. **p < .01. ***p < .001.

### Primary Analyses

**Figure 3:**
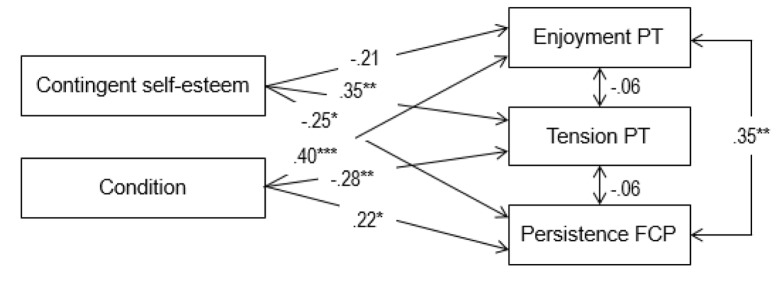
Structural Model Depicting the Relation between Contingent Self-esteem, Condition, Engagement and Disaffection (Study 2). *Note.* Standardized path coefficients are shown. Direct effects of self-esteem level on outcomes were estimated, but not shown for clarity purposes. Condition was coded as ‘0’ for the failure condition and as ‘1’ for the success condition. PT = Puzzle task; FCP = Free-choice period. *p < .05. **p < .01. ***p < .001.

We examined whether CSE and condition would predict enjoyment of and tension during the puzzle task and degree of persistence in a structural model. To do so, we allowed paths from CSE and condition to these three outcomes, while controlling for the level of self-esteem. This model was tested using MPlus 7 (Muthén & Muthén, 1998–2012). We corrected for the non-normality observed in some of the variables through robust maximum likelihood estimation (MLR). The model showed an excellent fit (χ²/ *df* = .91/ 2 = .46; CFI = 1.00; SRMR = .02; RMSEA = .00, 90% CI RMSEA = [.00, .19]). More specifically, as can be noticed in Figure 3, CSE related positively to tension during task execution, yet it related negatively to behavioral persistence during the free choice period. Additionally, condition related positively to both enjoyment and persistence, while being negatively related to tension.

To gain insight into the relation between CSE and the reasons for continued persistence we ran a second structural model with CSE predicting the different motives, while controlling for the level of self-esteem. This model had a perfect fit because it was fully saturated. Results showed that CSE related to introjection (β = .32, *p* < .01) and identified regulation (β = .29, *p* < .05), but was unrelated to intrinsic motivation (β = .12, *p* > .05) and external regulation (β = –.06, *p* > .05).

### Supplementary Analyses

To explore whether CSE related in a quadratic way to all outcomes, regression analyses were run wherein we controlled for a linear effect of CSE. Results showed that none of the quadratic terms related significantly to the outcomes (βs ranged from –.13 to .18; all *p*s > .05). We also examined whether the interactions between CSE and self-esteem and between CSE and condition were significant predictors of all outcomes in our main analyses. Results showed that none of the interactions were significant (βs ranged from –.06 to .23; all *p*s > .05), indicating that the effects of CSE on the outcomes were not moderated by condition and level of self-esteem.

### Brief discussion

The findings of Study 2 are partially consistent with those of Study 1, in spite of the use of an experimental rather than a correlation design and the study of the motivation and engagement dynamics at the situation instead of the domain level. Specifically, consistent with Study 1, individuals high in CSE reported more emotional disaffection during task execution, as reflected in their elevated tension. Different from Study 1, once the task was completed and participants were left by themselves, those high in CSE spent significantly less time on the puzzles, reflecting reduced behavioral engagement. Interestingly, to the extent individuals high in CSE did persist, they did so mostly for introjected (but also identified) rather than intrinsic reasons. So, in general, the pattern of findings associated with CSE was more pronounced negative and less ambiguous in Study 2, a pattern that was not altered depending on individuals’ level of self-esteem or the type of feedback they received (i.e., lack of interaction). Also, similar to Study 1, the effects of CSE on all outcomes were linear (instead of quadratic).

## General Discussion

Although an increasing number of studies has indicated the detrimental effects of CSE for individuals’ well-being (e.g., Lakey et al., 2014), less is known about the motivational correlates of CSE. Therefore, the general aim of this study was to examine the motivational and engagement correlates of CSE in achievement-related situations. Based on theoretical and limited empirical evidence, we hypothesized that individuals high in CSE would be highly committed to activities that signal high importance for their self-worth; yet, at the same time they may also feel emotionally more tense and conflicted about this activity which could hinder them in developing interest in the activity at hand. Therefore, we expected CSE to relate to a mixed pattern of motivational and engagement outcomes. Additionally, we explored whether CSE would be less harmful under certain conditions. To investigate these aims, we used data from both a cross-sectional and an experimental study.

### Contingent Self-esteem and Motivation

We first investigated the relation between CSE and a diversity of motivational subtypes. Although CSE related to all types of motives, the association with introjected regulation was most pronounced. This is consistent with previous theorizing (e.g., Deci & Ryan, 1995) asserting that individuals who hinge their self-esteem on achieving particular standards (e.g., achieving academically) are mainly carrying out the activity to comply with internal pressures. This effect emerged in Study 1, in which students’ “naturally occurring” motives for their school work were assessed as well as in Study 2 in which students engaged in a specific activity that was portrayed as carrying high diagnostic value for their competencies. Besides a link with introjection, we also found – at least in Study 1 – that students with higher academic CSE studied more out of external pressure. This is not surprising in light of the finding that CSE relates to psychologically controlling parenting (Wouters, Doumen, et al., 2013): Parents who are perceived to exert psychological control are assumed to make their children’s self-esteem and motivation more vulnerable for external and controlling influences.

Further, emphasizing the hypothesized motivational ambiguity of CSE, we also found that students who reported higher levels of academic CSE studied more because they found the study material to be personally relevant and even interesting and fun. Yet, the latter finding with respect to intrinsic motivation was not replicated in Study 2, as students with higher levels of global CSE did not persevere at the activity during the free-choice period out of sheer interest, but because they felt internally pressured or experienced the activity as personally relevant.

### Contingent Self-esteem and Engagement

As individuals high in CSE hinge their self-esteem on obtaining certain standards, it is reasonable to assume that they are highly committed and engaged to obtain these standards (Crocker, Luhtanen, et al., 2013; Lawrence & Williams, 2013). Nevertheless, we also hypothesized that these high levels of behavioral engagement would be accompanied by feelings of tension and reduced enjoyment. The relation between CSE and behavioral disaffection was examined in an explorative fashion, as it was unclear whether these feelings of tension would foster disaffection from activities at some moments. Results partially confirmed our hypotheses.

First, in both studies, CSE related positively to emotional disaffection, as indexed by a general measure of emotional disaffection and test anxiety in Study 1 and more felt tension during the puzzle task in Study 2. So, both studies clearly indicate that CSE is associated with maladaptive emotional states in achievement situations. This is important considering that maladaptive emotions in an achievement context, such as test anxiety, may have debilitating effects on students’ performance and learning, resulting in poor achievement or underachievement (Zeidner & Matthews, 2005).

Second, such a pattern did not occur for emotional engagement. Whereas individuals high in CSE did not report greater emotional engagement (as reflected by their enjoyment) during the execution of the puzzle task, in Study 1, CSE related positively to emotional engagement. This discrepancy may be explained by the context. In Study 1, students were asked about their emotional engagement with regard to learning, whereas in Study 2 emotional engagement pertained to the enjoyment of a task under pressuring circumstances. Possibly, individuals high in CSE enjoy learning in general, but this enjoyment may wane when learning is not the end-goal but achieving is. Future studies could further investigate the link between CSE and emotional engagement under diverse conditions, such as an evaluative (e.g., test) and non-evaluative environment. At least, the overall pattern with respect to emotional engagement and disaffection is an ambiguous one, as CSE related positively to emotional disaffection and, if anything, also positively to emotional engagement.

Third, with respect to behavioral indicators of engagement, whereas CSE related to *more self-reported* behavioral engagement in Study 1, it led to *less observed* behavioral engagement in Study 2. Future studies could further investigate this discrepant finding. We speculate on two possible explanations. First, it may be the case that persistence is inhibited for individuals high in CSE when they are confronted with a challenging task (such as the Tangram puzzle task) in an ego-involving situation, which may also trigger feelings of failure. Indeed, in both conditions participants were generally unable to perform the task successfully (average was about 3 and 1 for the success and failure condition, respectively). Thus, although individuals high in CSE may persist when they expect success, they may persist less when failure seems likely (Crocker & Park, 2003). Indeed, students who hinge their self-esteem on their academic achievements are more inclined to avoid challenges, especially if failure would come with a blow for their self-worth (Covington, 1984). Second, we should note that persistence in Study 2 was assessed during a free-choice period, while behavioral engagement in Study 1 pertained to the effort invested in school-related tasks. Perhaps, individuals high in CSE might especially disengage from the activity if they have the free choice to do so, that is, when nothing can be gained anymore.

Furthermore, Study 1 showed that the ambiguous effect of academic CSE on engagement runs at least partially through motivation. For instance, our results indicate that students who hinge their self-worth on their academic performance are more likely to study because they find it personally relevant which, in turn, makes them more likely to be academically engaged as well as less likely to be academically disaffected. However, we also found that students whose self-esteem is more dependent on how they perform academically were more likely to study because they felt pressured to study by their parents or other significant others which, in turn, was related to higher levels of behavioral and emotional disaffection.

### Additional Findings

Two additional findings deserve being highlighted. First, with regard to our explorative research question, results across both studies showed that the effect from CSE to the outcomes is linear and is not moderated by the level of self-esteem or the type of experience (i.e., success or fail experience). Taken together, this indicates that CSE relates to individuals’ functioning in an achievement-related situation independent of the general level of self-esteem or the valence of the situation (success or failure).

Second, at least in Study 1, identified regulation was more strongly related to engagement than intrinsic motivation. This finding is in line with previous studies showing a more pronounced role of identification for certain outcomes. Burton et al. (2006), for instance, examined the relation between intrinsic motivation, identified regulation, well-being, and academic performance among elementary school children. They found that intrinsic motivation for learning was the stronger correlate of well-being, whereas identified regulation was the stronger correlate of better grades. These and the current findings may be specifically due to the school context. Because many aspects and tasks in an educational context are not inherently interesting or enjoyable (Ryan & Deci, 2000), it would be more critical for children to come to fully endorse these non-interesting tasks, that is, to identify with its self-importance, which would then carry greater explanatory power for children’s school functioning.

### Limitations and Directions for Future Studies

This study had several limitations. First, although the sample size of Study 1 was sufficient, the sample size was relatively small in Study 2 which may have reduced the power of especially our interaction analyses. Second, although Study 2 employed a behavioral measure to assess persistence, all other constructs were assessed via self-reports. Future studies could assess the study variables in a broader sense, for example through assessing engagement in class as observed by the teacher. It would also be important to assess the relations between CSE, engagement, and motivation more dynamically with a longitudinal design as these relations could be reciprocal. Additionally, as we found some inconsistencies between the results of Study 1 and 2, more research is needed to determine why this was the case, for example by investigating both objective indicators and self-reports of behavioral engagement measured at the same level in one single study, or by assessing CSE at both the general and domain-specific level.

Second, especially with regard to Study 1, it seems that we are dealing with rather proximal mediators (Kenny & Judd, 2013): Our mediators are more closely related to CSE than to engagement outcomes. Hence, it may be interesting to identify other psychological mechanisms, such as the experience of psychological need satisfaction and need frustration (Bartholomew, Ntoumanis, Ryan, Bosch, & Thøgersen-Ntoumani, 2011), in future studies. Additionally, in Study 2, we only focused on motivation for persevering, thereby neglecting the diversity of reasons for not persevering (Green-Demers, Legault, Pelletier, & Pelletier, 2008). For example, participants could have stopped working on the puzzles to avoid failure and associated feelings of shame or because they found the puzzle task to be boring or not personally relevant.

Third, future studies could examine the generalizability of the current findings. For instance, the question can be raised whether a different experimental method to induce feelings of failure and success or threat versus non-threat, or the use of a different experimental task than the one used in Study 2 (i.e., Tangram Puzzle Task) may interact with CSE in the prediction of motivational and engagement dynamics. Further, the question can be raised whether observed correlates of CSE hold across different cultures. In this regard, Chen, Soenens, Vansteenkiste, Van Petegem, and Beyers (this issue) showed that parental guilt-induction (a facet of controlling parenting) was associated with less detrimental effects among Chinese adolescents compared to Belgian adolescents, although guilt-induction carried less desirable effects when compared to autonomy support among both groups of adolescents. As controlling parenting is positively related to CSE (Wouters, Doumen, et al., 2013), it might be the case that individuals from a collectivistic culture may be less negatively affected by higher levels of CSE.

A final issue that deserves further attention is the relation between CSE and level of self-esteem. Whereas level of self-esteem and CSE in the academic domain were slightly positively correlated in Study 1, level of self-esteem and CSE at the global level were moderately negatively associated in Study 2, with the latter finding being in line with previous studies (e.g., Wouters, Duriez, et al., 2013). Indeed, children or adolescents with lower global self-esteem may be more vulnerable to develop a fragile self-esteem which depends heavily on success and failure in general. However, our results also suggest that students who perceive themselves as less academically competent, are less likely to let their self-esteem depend on their achievements in the academic domain, as compared to students who feel more competent in this domain. Perhaps, when students feel less competent in a specific domain, they gradually devalue the domain or shift their attention to another domain to protect their self-esteem from failure in that domain (Crocker & Wolfe, 2001). Such strategies, however, may not apply when feeling unworthy in general. Future research is needed to explore these discrepant associations between level of self-esteem and CSE, depending on the level of assessment.

## Conclusion

Across two studies, CSE related to an ambiguous pattern of motivation and engagement. Although individuals high in CSE displayed higher levels of motivation in general, they were especially motivated because of introjected reasons. Further, CSE related positively to behavioral and emotional engagement in the domain of schooling in general, but to less behaviorally recorded persistence during a specific challenging situation. Across both studies, CSE was also associated with feelings of tension and anxiety. Overall, these findings highlight the ambiguity that characterizes the functioning of individuals high in CSE, both in terms of their engagement and motivational functioning.

## Footnotes

Twenty-nine students did not provide the name of their school or provided an incomplete name. Consequently, ICCs could only be calculated for maximum 612 students from 34 schools.

Testifying to the distinctiveness of the measures for academic CSE and introjection, the two-factor model (*N* = 626, χ² (19) = 152.88, RMSEA = .11, CFI = .93, SRMR = .07) showed a better fit than the one-factor model (*N* = 626, χ² (20) = 444.21, RMSEA = .18, CFI = .78, SRMR = .11; Δχ² (1) = 291.33, *p* < .001).

Based on the suggestion of a reviewer, we also analyzed a more general model by averaging across intrinsic motivation and identified regulation and across introjected and external regulation to generate two composite scores of, respectively, autonomous and controlled motivation. Additionally, we only focused on indicators of engagement and test anxiety in this model, thus omitting behavioral and emotional disaffection. In line with the results from our more specific models, academic CSE related positively to both autonomous and controlled motivation and, in turn, autonomous motivation related positively to both engagement indicators whereas controlled motivation related positively to test anxiety. Significant positive direct effects were found from academic CSE to behavioral and emotional engagement and test anxiety, but the direct effect on emotional engagement became non-significant when including both motivation types.

More specifically, in a parallel version of Model 1 with standardized continuous predictors, the interaction between academic CSE and academic SE on external regulation was significant. The interaction showed that the significant positive effect of academic CSE on external regulation was more pronounced for individuals scoring low on academic SE.

## Author Information

Sofie Wouters is a Postdoctoral Fellow of the Research Foundation – Flanders.
